# Associations between polypharmacy and potentially inappropriate medications with risk of falls among the elderly in Saudi Arabia

**DOI:** 10.1186/s12877-023-03852-y

**Published:** 2023-04-06

**Authors:** Khalid S. AlHarkan, Safaa Alsousi, Mujtaba AlMishqab, Majd Alawami, Jaffar Almearaj, Hassan Alhashim, Hassan Alamrad, Layla M. Alghamdi, Abdulelah Almansour, Reem S. AlOmar

**Affiliations:** 1 Department of Family and Community Medicine, College of Medicine, Imam Abdulrahman Bin Faisal University, Dammam, Saudi Arabia; 2 College of Medicine, Imam Abdulrahman Bin Faisal University, Dammam, Saudi Arabia

**Keywords:** Polypharmacy, Elderly, Falls, Potentially inappropriate medications

## Abstract

**Background:**

Falls are dangerous to the health of older adults and can impact their functional status leading to frailty. The use of potentially inappropriate medications (PIMs) among older adults may lead to adverse health outcomes and increase the risk of falls. Polypharmacy increases the incidence of falls. Beers criteria by the American Geriatric Society is one of the many criteria used to detect PIMs. It assesses the appropriateness of drug prescriptions (i.e., correct dose, duration, and indications) to ensure the safety of these drugs, reducing drug interactions and decreasing the hazards of side effects. This epidemiological study aims to explore the association between polypharmacy and Beers criteria with the risk of falls in the elderly.

**Method:**

A total of 387 outpatients aged 60 or older were interviewed in person. The patients were recruited from the University Hospital and the Family and Community Medicine Center in Khobar city, Saudi Arabia, between the period of November 2021 to March 2022. All patients were able to walk independently. The survey began by collecting patients’ demographics, gathering medication history, and asking three key questions to detect the risk of falls which was developed by the Center of Disease Control (CDC). Polypharmacy (defined as concurrent use of five or more medications) and PIMs (defined as use of one or more medications in the Beers list) were examined against risk of falls in the elderly. Multiple logistic regression analyses were used to estimate adjusted Odds Ratios (ORs).

**Result:**

A total of 387 patients participated in the study; 62% were male, and most participants belonged to the 60 < 65 years age category (47.80%). Among all patients, 55% had a high risk of falling, and 21% of patients had fell during the past year. Polypharmacy applied to 50.90% of all patients, while Beers criteria positive group applied to 51.42%. Risk of falls and prior falls were associated with polypharmacy both before and after adjustment.

**Conclusion:**

The results showed a significant association between risk of falls with polypharmacy and PIMs, and more than half of our study population had a high risk of falls. Of those at a higher risk, one out of five had indeed experienced a fall in the last 12 months. Higher rates of falls were associated with older aged patients, lower educational levels, female gender, and cardiovascular medications.

## Introduction

Falls are dangerous to the health of older adults and can impact their functional status, leading to frailty [1]. A fall can be described as an event that results in the falling of a person to a lower level or the ground, that is not due to a major intrinsic event (such as a stroke) or overwhelming hazard [2]. Age is one of the risk factors for falls, especially above 65 years old. In the United States (US), one out of four adults aged 65 and older reported falling, resulting in about 36 million falls each year. These falls cost $50 billion annually [1]. In Riyadh city, Saudi Arabia, the prevalence of falls in the elderly was 50%, and 74% of them had injuries as a result of that fall; these injuries range between fractures to bruises, and around half of them needed a walking aid after the fall [3]. Falls are the main cause of fatal and nonfatal injuries among older adults [4]. The main risk factors that lead to falls were neurological diseases, musculoskeletal problems, psychosocial, polypharmacy, and many others [5]. In Unaizah city, Saudi Arabia, prevalence of falls was 31%, and more than half of the study population had polypharmacy [6].

Polypharmacy is a state in which five medications, or more, are consumed concurrently [5]. The use of potentially inappropriate medications (PIMs) among older adults may lead to adverse health outcomes and increases the risk of falls [7, 8]. Beers criteria is one of the many criteria used to detect PIMs. It is the most common list used to describe inappropriate drugs for older adults and was recently updated in 2019 by the American Geriatrics Society [7]. It assesses the appropriateness of drug prescription guidelines (i.e., correct dose, duration, and indications) to ensure the safety of these drugs, decrease the hazards of side effects, and reduce drug interactions [7].

To our knowledge, there are limited local publications that have explicitly addressed the relation between polypharmacy and frequent falls in elderly patients. Therefore, this study aims to explore the association between polypharmacy and Beers criteria with the risk of falls in the elderly.

## Methods

### Study design and settings

This cross-sectional study was conducted at the Family and Community Medicine Centre at Imam Abdulrahman bin Faisal University (IAU) and King Fahad Hospital of the University (KFHU) outpatient departments in Khobar city, Saudi Arabia, between the period of November 2021 to March 2022. This study was approved by the Institutional Review Board of Imam Abdulrahman bin Faisal University (IRB-UGS-2021-01-350). Written informed consents were obtained from all patients before interviewing them.

### Sample size and sampling technique

The minimum required sample size was calculated to be 384. This was based on a prevalence of 49.9% of falls among the Saudi elderly in 2018 [3]. With a precision of 5% and at an alpha level of 0.05. Sample size calculation was performed in Epi info 7.0. A systematic sampling technique was used to select the study participants who were approached by six research assistants. This was done by obtaining the patient lists from the outpatients’ department and choosing every 4th patient.

All patients were able to walk independently. Patients who had acute illness or were diagnosed with neurocognitive disorders, such as dementia, and Parkinson’s disease, and/or those who cannot recall their medications, were excluded. A total of 393 patients were approached; however, 4 were excluded due to having dementia and another 2 were excluded due to Parkinson’s disease. Hence, a total of 387 patients were included after application of the exclusion criteria.

### Demographics and risk of fall assessment

The survey began by obtaining the patients’ sociodemographic information. These included age (60 < 65, 65 < 70, 70 < 75, 75 < 80, 80 < 85 and ≥ 85 years old), gender (males, females), marital status (single, married, divorced, widowed), living situation (alone or with family), and educational level (illiterate, elementary school, middle school, high school, university education).

The assessment for risk of falls was based on Stopping Elderly Accidents, Deaths, and Injuries (STEADI), which was developed by the Center of Disease Control (CDC) [9]. The three key questions asked were whether the patient feels unsteady when standing or walking, whether the patient worried about falling, and if the patient had fallen during the previous year. In case the answer to one of those three questions was yes, then the patient is considered at a high risk of falls. For the last question, however, if the answer was yes, then two additional questions were asked. The first was how many times he/she fell, and the second was whether there were any related injuries to the fall.

### Medication assessment

A detailed medication history was obtained with the help of the patients’ caregivers. We classified patients based on the number of medications into two groups: polypharmacy (concurrent five medications or more) versus non-polypharmacy (less than concurrent five medications).

Furthermore, we classified patients based on the Beers criteria for potentially inappropriate medications into two groups: positive Beers criteria (patients who had one medication in the Beers list or more) versus negative Beers criteria.

We further grouped medications into cardiovascular medications, such as ACE inhibitors; angiotensin receptor blockers (ARB), calcium channel blockers, beta blockers, and antiarrhythmics; anticoagulants such as Warfarin, low molecular weight heparin, and direct oral anticoagulants; antiplatelets, such as aspirin, clopidogrel, ticlopidine, and ticagrelor; endocrinology medications, such as anti-diabetes mellitus drugs, vitamin D, thyroid, and osteoporosis drugs; dyslipidemia medications, such as statin; gastroenterology medications, such as proton pump inhibitors (PPIs), laxatives, anti-emetics, and inflammatory bowel disease drugs; diuretics, such as Furosemide; neurology medications, such as anti-Parkinson’s drugs, and anti-seizure drugs; psychiatric medications, such as anti-depressants and antipsychotics; respiratory inhalers; and urology medications, such as anti-BPH drugs [10].

### Statistical analysis

All analyses were performed using Stata software version 15.0. The main outcome of the study was the risk of falls (low/high), and the secondary outcome was the occurrence of prior falls in the last year. Descriptive statistics were reported as frequencies and percentages. Two sets of analyses were performed; the first was to assess the risk of falling and polypharmacy, and the second was to assess the risk of falling and the Beers criteria groups, all while adjusting for confounding variables. Bivariate analyses were computed through Chi-squared (**χ**^2^) tests. Trends of proportions over age were tested for statistical significance. Unadjusted and adjusted odds ratios (ORs) and their 95% confidence intervals (CIs) were computed through a series of binary logistic regression analyses. Choice of variables in the final model was based on a Directed Acyclic Graph of the associations and the best model fit. All levels of significance were set at 0.05.

## Results

### Sociodemographic and fall-related characteristics of participants

A total of 387 patients participated in the study. Overall, there were 62.53% males and 37.47% females. Most participants belonged in the 60 < 65 years age category (47.80%), and only 4.95% were aged above 85 years. Over 95% lived with their families, and 36.18% were university graduates, while 17.31% were illiterate. With regards to marital status, 1.03% were single and 78.55% were married (Table 1). Among all patients, 48.84% reported feeling unsteady when walking, while 32.04% worry about falling. Over 21% of patients fell during the past year. Among them, 11 (2.84%) fell four or more times in the past year, and 54.22% reported an injury due to that fall. Hence, a high risk of falls was found to be present in 55.50% of all participants (Table 2).

**Table 1:** Descriptive statistics of study participants

Characteristic	N (%) 387 (100.00%)
**Age**	
60 < 65 years	185 (47.80)
65 < 70 years	80 (20.67)
70 < 75 years	60 (15.50)
75 < 80 years	28 (07.24)
80 < 85 years	15 (03.88)
≥ 85 years	19 (04.91)
**Gender**	
Males	242 (62.53)
Females	145 (37.47)
**Living situation**	
Alone	17 (04.39)
With family	370 (95.61)
**Educational status**	
Illiterate	67 (17.31)
Elementary school	79 (20.41)
Middle school	36 (09.30)
High school	65 (16.80)
University	140 (36.18)
**Marital status**	
Single	4 (01.03)
Married	304 (78.55)
Widowed	74 (19.12)
Divorced	5 (01.29)

**Table 2:** Fall related characteristics of study participants

Characteristic	N (%) 387 (100.00%)
**Feels unsteady when walking**	
No	198 (51.16)
Yes	189 (48.84)
**Worries about falling**	
No	263 (67.96)
Yes	124 (32.04)
**Has fallen in the past year**	
No	304 (78.55)
Yes	83 (21.45)
**Number of times fallen**	
Never	304 (78.55)
Once	44 (11.37)
Twice	20 (05.17)
Three times	8 (02.07)
Four or more times	11 (02.84)
**If fallen, injured**	
No	38 (45.78)
Yes	45 (54.22)
**Risk of falls**	
Low	173 (44.70)
High	214 (55.30)

### Associations between sociodemographic variables and the risk of falls and prior falls

Both the risk of falls and prior falls were found to be statistically significantly associated with age (P < 0.001 and P = 0.04 respectively). The percent of patients who are at a high risk of falls is seen to increase with the increase in age (P for trend < 0.001). Gender was also highly statistically significant with both risk of falls and prior falls with a higher percent observed among females (P < 0.001). Significant associations were also observed for educational status and marital status. No association was observed for the living situation (Table 3).

**Table 3:** Associations between sociodemographic characteristics with the risk of falls and prior falls

Characteristic	Risk of falls		Prior falls	
	Low N (%) 173 (44.70)	High N (%) 214 (55.30)	No N (%) 304 (78.55)	Yes N (%) 83 (21.45)
^‡^ **Age**				
60 < 65 years	101 (54.59)	84 (45.41)	155 (83.78)	30 (16.22)
65 < 70 years	35 (43.75)	45 (56.25)	64 (80.00)	16 (20.00)
70 < 75 years	25 (41.67)	35 (58.33)	45 (75.00)	15 (25.00)
75 < 80 years	25 (41.67)	21 (75.00)	19 (67.86)	9 (32.14)
80 < 85 years	7 (25.00)	12 (80.00)	9 (60.00)	6 (40.00)
≥ 85 years	2 (10.53)	17 (89.47)	12 (63.13)	7 (36.84)
*P-value*	*< 0.001*		*0.04*	
**Gender**				
Males	130 (53.72)	112 (46.28)	208 (85.95)	34 (14.05)
Females	43 (29.66)	102 (70.34)	96 (66.21)	49 (33.79)
*P-value*	< 0.001		< 0.001	
**Living situation**				
Alone	6 (35.29)	11 (64.71)	11 (64.71)	6 (35.29)
With family	167 (45.14)	203 (54.56)	293 (79.19)	77 (20.81)
*P-value*	0.42		0.15	
^‡^ **Educational status**				
Illiterate	15 (22.39)	52 (77.61)	41 (61.19)	26 (38.81)
Elementary school	33 (41.77)	46 (58.23)	64 (81.01)	15 (18.99)
Middle school	15 (41.67)	21 (58.33)	28 (77.78)	8 (22.22)
High school	25 (38.46)	40 (61.54)	50 (76.92)	15 (23.08)
University	85 (60.71)	55 (39.29)	121 (86.43)	19 (13.57)
*P-value*	< 0.001		0.002	
**Marital status**				
Single	2 (50.00)	2 (50.00)	4 (100.00)	0
Married	152 (50.00)	152 (50.00)	249 (81.91)	55 (18.09)
Widowed	18 (24.32)	56 (75.68)	47 (63.51)	27 (36.49)
Divorced	1 (29.00)	4 (80.00)	4 (80.00)	1 (20.00)
*P-value*	< 0.001		0.004	

^*‡*^ *P for trend < 0.001 for risk of falls*

### Associations between medication use and the risk of falls and prior falls

**Fig. 1:**
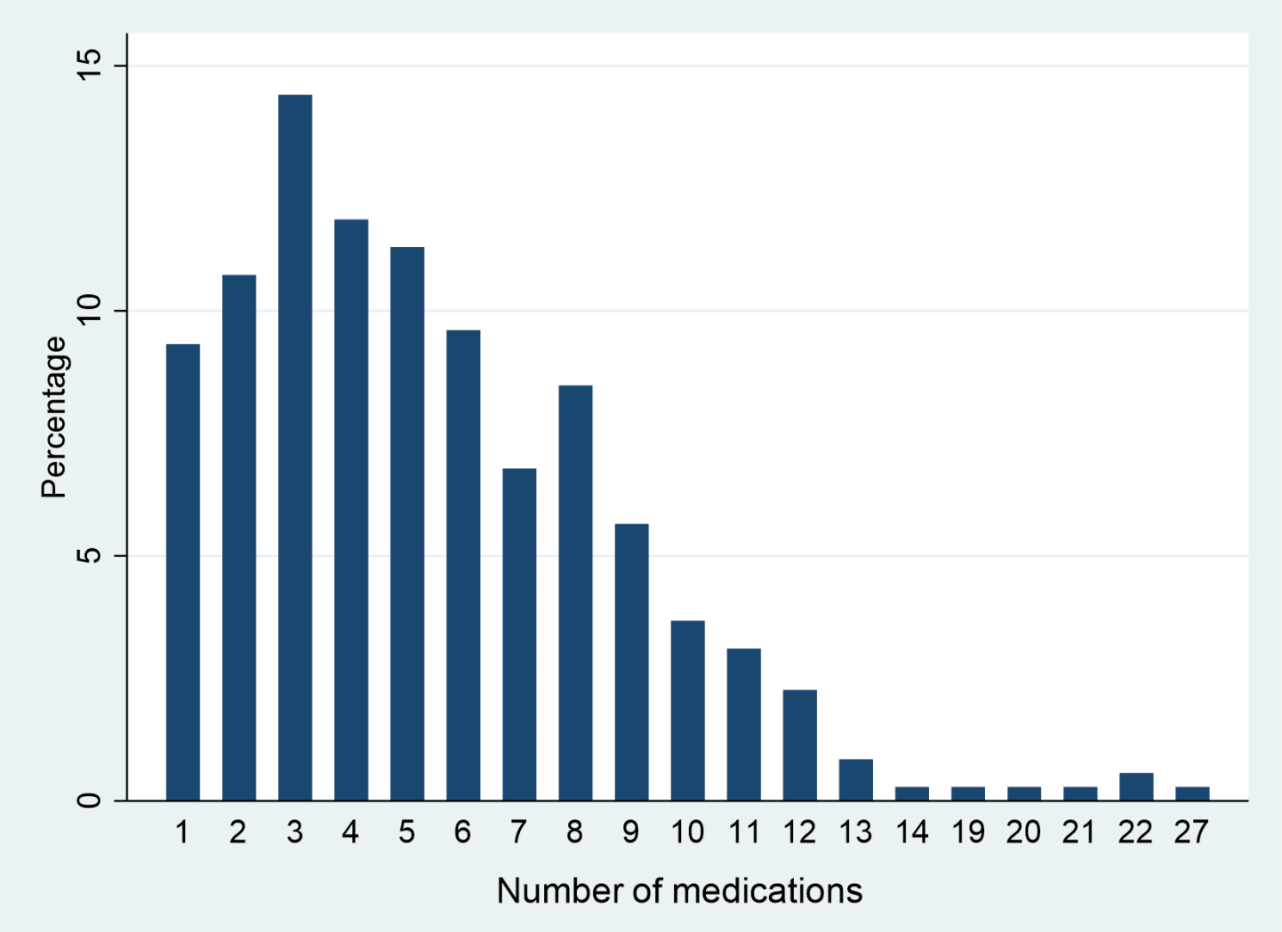
Number of prescribed medications to study participants

Figure 1 shows the number of prescribed medications to all patients, where a significant number of patients consume over 15 medications a day. Polypharmacy applied to 50.90% of all patients, while Beers criteria applied to 51.42%. Examining specific medication uses, cardiovascular medications were the most frequently reported (53%), followed by endocrine medications (46%) and dyslipidemia (35%), as shown in Table 4.

**Table 4:** Associations between specific drug class, polypharmacy, and Beers criteria with risk of falls

Medications	N (%) 387 (100.00%)	Risk of fall		P-value
		Low	High	
**Cardiovascular**				0.006
No	180 (46.51)	94 (52.22)	86 (47.78)	
Yes	207 (53.49)	79 (38.16)	128 (61.84)	
**Anticoagulants**				0.42
No	370 (95.61)	167 (45.14)	203 (54.86)	
Yes	17 (04.39)	6 (35.29)	11 (64.71)	
**Antiplatelets**				0.001
No	274 (70.80)	138 (50.36)	136 (49.64)	
Yes	113 (29.20)	35 (30.97)	78 (69.03)	
**Endocrine**				0.55
No	206 (53.23)	95 (46.12)	111 (53.88)	
Yes	181 (46.77)	78 (43.09)	103 (56.91)	
**Dyslipidemia**				0.002
No	249 (64.34)	126 (50.60)	123 (49.40)	
Yes	138 (35.66)	47 (34.06)	91 (65.94)	
**Gastrointestinal**				< 0.001
No	314 (81.14)	157 (50.00)	157 (50.00)	
Yes	73 (18.86)	16 (21.92)	57 (78.08)	
**Diuretics**				0.003
No	325 (83.98)	156 (48.00)	169 (52.00)	
Yes	62 (16.02)	17 (27.42)	45 (72.58)	
**Neurology**				0.20
No	385 (99.48)	173 (44.94)	212 (55.06)	
Yes	2 (00.52)	0	2 (100.00)	
**Inhalers**				0.07
No	373 (96.38)	170 (45.58)	203 (54.42)	
Yes	14 (03.62)	3 (21.43)	11 (78.57)	
**Urology**				0.06
No	365 (94.32)	159 (43.56)	206 (56.44)	
Yes	22 (05.68)	14 (63.64)	8 (36.36)	
**Psychiatric**				0.88
No	385 (99.48)	172 (44.68)	213 (55.32)	
Yes	2 (0.52)	1 (50.00)	1 (50.00)	
**Polypharmacy**				< 0.001
No	197 (50.90)	120 (60.91)	77 (39.09)	
Yes	190 (49.10)	53 (27.89)	137 (72.11)	
**Beers criteria**				0.002
No	188 (48.58)	99 (52.66)	89 (47.34)	
Yes	199 (51.42)	74 (37.19)	125 (62.81)	

According to polypharmacy and Beers criteria group of patients, statistically significant associations were found with risk of falls (P < 0.001) and (P = 0.002), respectively. Although a higher percent of prior falls had happened among participants who were on polypharmacy and who were positive for Beers criteria, only polypharmacy was statistically significant (P < 0.001).

### Multivariate associations between polypharmacy and risk of falls and prior falls

Table 5 shows the adjusted ORs and their respective 95% CIs. Polypharmacy was found to significantly increase the risk of falls and prior falls after adjustment for age, gender, living situations, and educational status. After adjustment, the odds of a high risk of falling were greater for polypharmacy patients compared to those who are not (OR = 3.62, 95% CI = 2.05–5.19, P-value < 0.001), and the OR of prior falls was 2.83 (95% CI = 1.59–5.04). Increasing age was also a factor for the risk of falls; however, only patients who belonged to the ≥ 85 years category were significant (Adjusted OR = 7.67, 95% CI = 1.61–36.55). The risk of prior falls for females were higher than males (OR = 2.07, 95% CI = 1.14–3.77).

**Table 5:** Adjusted Odds Ratios for the risk of falls and prior falls in relation to polypharmacy

Predictors	Risk of falls		Prior falls	
	OR	95% CI	OR	95% CI
**Polypharmacy**				
No	*Ref			
Yes	**3.62**	**2.05–5.19**	**2.83**	**1.59–5.04**
**Age**				
60 < 65 years	Ref			
65 < 70 years	1.52	0.86–2.70	1.24	0.61–2.53
70 < 75 years	1.66	0.87–3.16	1.61	0.76–3.42
75 < 80 years	2.52	0.95–6.67	1.73	0.66–4.53
80 < 85 years	2.47	0.60–10.04	1.98	0.56–6.93
≥ 85 years	**7.67**	**1.61–36.55**	1.99	0.66–6.00
**Gender**				
Males	Ref			
Females	1.58	0.93–2.69	**2.07**	**1.14–3.77**
**Living situation**				
Alone	1.04	0.31–3.43	1.26	0.38–4.15
With family	Ref			
**Educational status**				
Illiterate	2.23	0.99–4.99	1.50	0.63–3.53
Elementary school	1.44	0.76–2.70	0.92	0.41–2.06
Middle school	1.86	0.83–4.17	1.43	0.54–3.81
High school	**2.40**	**1.25–4.61**	1.73	0.78–3.81
University	Ref			

*Reference

### Multivariate associations between Beers criteria and risk of falls and prior falls

Table 6 shows the adjusted ORs and their respective 95% CI and P-values. Beers criteria patients were found to be at an increased risk of falls after adjustment (OR = 1.86, 95% CI = 1.19–2.90 respectively). Increasing age was a predictor, where the higher the age the higher the odds of falls, reaching 7.97 (95% CI = 1.71–37.12) for patients belonging to the ≥ 85 years category. Females were at a significant risk of falls and prior falls (OR = 2.09, 95% CI = 1.26–3.49 and OR = 2.57, 95% CI = 1.44 and 4.59, respectively). For educational status, compared to university graduates, those with up to a high school education were at a higher risk, although only significant for illiterate patients and patients with a high school degree. Age, living situation, and educational status were not associated with prior falls.

**Table 6:** Adjusted Odds Ratios for the risk of falls in relation to Beers criteria

	Risk of falls		Prior falls	
	OR	95% CI	OR	95% CI
**Beers criteria**				
No	*Ref			
Yes	**1.86**	**1.19–2.90**	1.22	0.72–2.07
**Age**				
60 < 65 years	Ref			
65 < 70 years	1.38	0.79–2.42	1.21	0.60–2.43
70 < 75 years	1.57	0.84–2.93	1.63	0.78–3.41
75 < 80 years	2.61	1.01–6.74	1.82	0.70–4.72
80 < 85 years	2.77	0.69–11.03	2.36	0.69–8.05
≥ 85 years	**7.97**	**1.71–37.12**	2.26	0.77–6.63
**Gender**				
Males	Ref			
Females	**2.09**	**1.26–3.49**	**2.57**	**1.44–4.59**
**Living situation**				
Alone	1.06	0.33–3.39	1.22	0.38–3.87
With family	Ref			
**Educational status**				
Illiterate	**2.36**	**1.07–5.19**	1.68	0.72–3.88
Elementary school	1.48	0.80–2.73	1.01	0.45–2.32
Middle school	2.06	0.94–4.50	1.61	0.62–4.19
High school	**2.33**	**1.23–4.39**	1.72	0.78–3.75
University	Ref			

*Reference

## Discussion

The present study assessed the risk of falls in the elderly using the CDC’s STEADI tool and examined its association with polypharmacy and PIMs. The primary findings were that over half the participants were at a high risk of falls and that the odds of a high risk of falls were higher among patients who were on polypharmacy and patients who were positive for Beers criteria. The number of medications is one of the major factors that may contribute to the risk of falls. In our study, the average number of medications is 5.1, and only 6 patients (1.5%) of the study population consume over 15 medications [11]. One patient in our study population reported using 27 medications, which has been observed in our clinical practice due to lack of primary care physicians for continuous monitoring of overall health and the patient’s ability to get multiple prescriptions by different providers. Over half of our study population reported use of cardiovascular medications, such as ACE inhibitors, angiotensin receptor blocker (ARB), calcium channel blockers, beta blockers, and antiarrhythmics. Similar to our findings, one national study found an average number of medications for the elderly was 6.4 [12].

One study found that out of 116 patients, 109 of them were taking more than 4 medications during the time they experienced a fall. They also found a 14% increase in the risk of falls with the addition of each medication regardless of drug class. Furthermore, a systematic review and meta-analysis examining polypharmacy in the geriatric population estimated a 6% increase in the risk of falls with the addition of each medication [13].

The main class of medications associated with risk of falls in this current study were cardiovascular medications (antihypertensives and antiarrhythmics). Consistent with our findings, a previous study of elderly patients aged 66 years and older showed that an increased risk of injurious falls occurred after starting antihypertensive medications [14]. Another large study of approximately 5,000 community-dwelling elderlies aged 70 years and older suggested that antihypertensive drugs were linked with an increased risk of fall injuries, particularly in those with a history of falls [15]. An increase in the risk of falls may be due to the fact that such medications may cause bradycardia, postural hypotension, edema of lower extremities, and dizziness—all of which may lead to balance and gait impairments and result in a fall or fall-related injury [15–17]. On the other hand, a community study of 598 elderly patients with hypertension found that high doses of antihypertensive agents were not associated with an increased risk for falls [18]. However, there are inherent differences between this study especially in terms of the method of data collection, which was based on an interview rather than using an electronic prescription service.

Gastrointestinal medications were also associated with the risk of falls. The most common medication within this category was PPI and several studies have suggested that PPIs increase the risk of falls [19–23]. Moreover, one study not only suggested that the use of PPI increases the risk of falls, but fractures too [23]. One of the possible explanations is that PPIs are linked with frailty which may increase the risk of falling. PPIs were also linked with osteoporotic type fractures and low bone mineral density compared to patients who do not use PPIs [20]. Another possible contributing factor is that PPIs decrease B12 absorption, which in the long term leads to demyelinating neurologic diseases, gait disorders, muscle weaknesses, cognitive impairments, and visual problems—all of which can cause falls [21, 23].

Age and gender are known to be major risk factors for falls. According to a Canadian study, the prevalence of falls increased with age. Our results showed a significant p for trend with increasing age, and we found that the ≥ 85-year age group have a higher risk of falls compared to the younger age group of 60 to 65 year olds. The higher risk of falls in the older age group could be explained by low muscular mass, visual and hearing impairment, balance disorders, and comorbidities, which furtherly increase the need for polypharmacy [24]. As for gender, similar to our results, a study showed that females were at a threefold increased risk of falls when compared to males, which can be explained by many factors, such as decreased muscle mass, use of high heels, and accidents when working at home [25, 26]. It should be noted however, that the number of patients within the ≥ 85-year age group was very small, hence may have attributed to the extreme high-risk estimate.

A study by Almegbel et al., in 2018 found that patients with high educational levels were at a lower risk of experiencing a fall [3]. This may be due to the awareness of the highly educated people about fall preventive measures and home hazards, and perhaps that group is better at following prescribed drug regimens [27].

This study has several limitations, one of them is that patients were asked to recall details about the fall event, which introduces recall bias. Also, we did not specifically ask about the duration of the medication usage nor take into consideration drug interactions.

## Conclusion

The results showed a significant association between the risk of falls and both polypharmacy and PIMs. More than half of our study population had an elevated risk of falls. Of those at a higher risk, one out of five had indeed experienced a fall in the last 12 months. Higher rates of falls were associated with older age, lower educational levels, female gender, and cardiovascular medications. Routine assessment of the risk of falls are highly recommended, as well as integration of the Beers criteria into electronic medical records to highlight potentially inappropriate medications in this vulnerable age group.

## Data Availability

The dataset used and analysed during the current study is available from the corresponding author upon reasonable request.

## Abbreviations

PIMs: Potentially inappropriate medications
PPI: Proton pump inhibitors
ACE: Acetylcholinesterase
ARB: Angiotensin receptor blocker
STEADI: Stopping Elderly Accidents, Deaths, and Injuries
CDC: Center of Disease Control
BPH: Benign Prostatic Hyperplasia
